# Response of human chondrocytes and mesenchymal stromal cells to a decellularized human dermis

**DOI:** 10.1186/1471-2474-14-12

**Published:** 2013-01-07

**Authors:** Gianluca Giavaresi, Elena Bondioli, Davide Melandri, Roberto Giardino, Matilde Tschon, Paola Torricelli, Giovanna Cenacchi, Roberto Rotini, Alessandro Castagna, Francesca Veronesi, Stefania Pagani, Milena Fini

**Affiliations:** 1 Laboratory of Preclinical and Surgical Studies, Rizzoli Orthopaedic Institute IRCCS, Bologna, Italy; 2 RIT Department, Laboratory of Biocompatibility, Innovative Technologies and Advanced Therapies, Rizzoli Orthopaedic Institute, Bologna, Italy; 3 Burn Intensive Care Unit and “Regione Emilia Romagna” Skin Bank, Bufalini Hospital, Cesena, Italy; 4 Department of Biomedical and Neuromotor Sciences, Saint Orsola Malpighi Hospital, Bologna, Italy; 5 Shoulder and Elbow Surgery, Rizzoli Orthopaedic Institute, Bologna, Italy; 6 Shoulder Surgery, Istituto Clinico Humanitas IRCCS, Milan, Italy

**Keywords:** Articular chondrocytes, Mesenchymal bone marrow stromal cells, Decellularized dermis, Bioactivity, *In vitro* study, Cartilage tissue engineering

## Abstract

**Background:**

Although progress has been made in the treatment of articular cartilage lesions, they are still a major challenge because current techniques do not provide satisfactory long-term outcomes. Tissue engineering and the use of functional biomaterials might be an alternative regenerative strategy and fulfill clinical needs. Decellularized extracellular matrices have generated interest as functional biologic scaffolds, but there are few studies on cartilage regeneration. The aim of this study was to evaluate *in vitro* the biological influence of a newly developed decellularized human dermal extracellular matrix on two human primary cultures.

**Methods:**

Normal human articular chondrocytes (NHAC-kn) and human mesenchymal stromal cells (hMSC) from healthy donors were seeded in polystyrene wells as controls (CTR), and on decellularized human dermis batches (HDM_derm) for 7 and 14 days. Cellular proliferation and differentiation, and anabolic and catabolic synthetic activity were quantified at each experimental time. Histology and scanning electron microscopy were used to evaluate morphology and ultrastructure.

**Results:**

Both cell cultures had a similar proliferation rate that increased significantly (*p* < 0.0005) at 14 days. In comparison with CTR, at 14 days NHAC-kn enhanced procollagen type II (CPII, *p* < 0.05) and aggrecan synthesis (*p* < 0.0005), whereas hMSC significantly enhanced aggrecan synthesis (*p* < 0.0005) and transforming growth factor-beta1 release (TGF-β1, *p* < 0.0005) at both experimental times. Neither inflammatory stimulus nor catabolic activity induction was observed. By comparing data of the two primary cells, NHAC-kn synthesized significantly more CPII than did hMSC at both experimental times (*p* < 0.005), whereas hMSC synthesized more aggrecan at 7 days (*p* < 0.005) and TGF-β1 at both experimental times than did NHAC-kn (*p* < 0.005).

**Conclusions:**

The results obtained showed that in *in vitro* conditions HDM_derm behaves as a suitable scaffold for the growth of both well-differentiated chondrocytes and undifferentiated mesenchymal cells, thus ensuring a biocompatible and bioactive substrate. Further studies are mandatory to test the use of HDM_derm with tissue engineering to assess its therapeutic and functional effectiveness in cartilage regeneration.

## Background

Articular cartilage lesions following acute trauma or pathological conditions such as osteonecrosis, osteochondritis and rheumatoid arthritis are responsible for the highest rate of disability worldwide [1]. These pathologies represent a serious socio-economic problem for the patient with regards to morbidity, gait abnormality, pain and inability to return to work and for health systems due to the high related costs. Present treatment methods do not provide a satisfactory long-term outcome; an innovative and widely investigated approach consists of grafting scaffold alone or immature tissue to allow chondrogenesis to occur *in situ*[2,3]. Functional biomaterials for cartilage regeneration should ideally provide (1) specific biomimetic hierarchical structures that might result in the formation of hyaline cartilage; (2) mechanical compatibility that could maintain original biomaterial morphology under repetitive physiological loads; (3) release of biosignals to promote chondrogenesis differentiation; and (4) enhanced integration properties with host tissues, thus allowing host cell infiltration and ideally even enhance the biology of healing [4].

Xenogenic and allogenic decellularized extracellular matrices (ECMs) have aroused great interest as functional biologic scaffolds also in cartilage tissue regeration [1,5]. ECMs elicit distinct host-tissue histological and morphologic responses, depending on the species of origin, tissue of origin, processing methods, and/or method of terminal sterilization [6]. They provide a compromise between biomechanical and biological function for the healing process, modulating host cell response and, consequently, accelerating the biology of tissue repair and integration with adjacent cartilage [6,7].

To the authors’ knowledge, there are few studies that assess the biological properties of acellular ECM membranes as scaffolds for cartilage tissue engineering and regenerative medicine, not only in terms of proliferation and viability, but also anabolic and catabolic synthetic activity [8-13]. Yang Q et al. and Yang Z et al. developed a natural acellular 3D interconnected porous scaffold which resulted in a valid support for the attachment, proliferation and differentiation of bone marrow mesenchymal stromal cells into chondrocytes. Gong et al. proposed a sandwich model of an acellular cartilage sheet for *in vitro* and *in vivo* cartilage engineering, thus mimicking the native environment and the structure of cartilage. Finally, a previous study by the current authors on the behaviour of a decellularized human dermis (HDM_derm), cultured with primary rat tenocytes, showed *in vitro* a high biological performance and mechanical competence of HDM_derm [14].

The aim of the present study was to evaluate *in vitro* the biological influence of this newly developed decellularized human dermal ECM on two human primary cultures - normal human articular chondrocytes (NHAC-kn) derived from knee articular cartilage, which represents the mature phenotype of hyaline articular cartilage, and human mesenchymal stromal cells (hMSC) derived from bone marrow.

## Methods

### Decellularization of human dermis

Decellularized human dermis (HDM_derm) was taken from the back of four multi-organ and/or multi-tissue donors by following strictly Directive 2004/23/EC and Italian Transplant Center Guidelines on harvesting, processing and distributing tissues for transplantation.

In sterile conditions, using an electric dermatome, the epidermis was separated from the dermis trunk area and dermis samples of about 10 cm^2^ were dissected. The thickness of the dermal samples was about 0.8 mm. The dermis grafts were rinsed with 0.9% NaCl solution and stored in this solution for transport (2-4°C) to the treatment station (< 12 hrs) where they were submitted aseptically to a combined treatment of decellularization. The tissue was treated with 2.5% trypsin (GIBCO, catalogue n. 15090) and placed in an incubator overnight (5% CO_2_ at 37°C), washed twice with sterile 0.9% NaCl to remove trypsin remnants, then dipped in RPMI medium (containing 10000 IU/ml penicillin, 10 mg/ml streptomycin, 25 μg/ml amphotericin B) for 15 minutes and sealed inside cryofreezing bags (Hemofreeze bag – DF 700–3 by Fresenius Kabi) without adding cryoprotectors. Finally, the dermis was submitted to irradiation with γ rays (100 Gy) and stored in nitrogen vapour at -180/-190°C. This method provides a decellularized ECM scaffold, as shown by histology (H&E, DAPI stain), Transmission Electron Microscopy, cell viability test and DNA amount (Picogreen DNA Assay) [6].

### Cell proliferation and biosynthesis on decellularized human dermis

Normal human articular chondrocytes (NHAC-kn, Clonetics™ Cell System, BioWhittaker Italia, Caravaggio, Italy), derived from a single-donor knee articular cartilage, and human mesenchymal stromal cells (Poietics™ hMSC, Lonza Walkersville Inc., Walkersville, USA) harvested and cultured from bone marrow of a single donor, were used for the experiment. NHAC-kn and hMSC cells were expanded using the Chondrocyte Growth Medium (CGM, containing fetal bovine serum 5%, gentamicin sulphate-amphotericin B 0.1%, b-Fibroblast Growth Factor 0.5%, R3-Insulin-like Growth Factor-1 0.2%, insulin 0.2%, transferrin 0.1%) and the Mesenchymal Stem Cell Growth Medium (MSCGM, containing 10% FCS, 30 μg/ml Gentamicin and 15 ng/ml Amphotericin, Lonza Walkersville Inc., Walkersville, USA). Four different batches of HDM_derm were aseptically cut into 10 × 10 mm^2^ pieces; NHAC-kn, at passage 1, or hMSC were seeded onto forty-eight specimens at a density of 1 × 10^5^ cells. The same concentration of NAHC-kn and hMSC cells was seeded in empty polystyrene wells as control (CTR). After 24 h incubation, of growth media were changed with Chondrocyte Differentiation Medium (CDM, containing fetal bovine serum 5%, gentamycin sulphate-amphotericin B 0.1%, Transforming Growth Factor β-3 0.5%, R3- Insulin-like Growth Factor-1 0.2%, insulin 0.2%, transferrin 0.2%, and ascorbic acid 2.5%) in NHAC-kn cultures and with Complete Chondrocyte Induction Medium (Lonza Walkersville Inc., Walkersville, USA) in hMSC cultures. After 24 h of incubation, the medium was collected and non-adherent cells were counted. Cell adhesion was calculated as the difference between seeded cells and non-adherent cells, present in the medium and attached to the 24-well plate bottom, expressed as a percentage of the number of the seeded cells. At 7 and 14 days, the supernatant was collected and the following tests were performed: procollagen type II C-propeptide (CPII ELISA, IBEX Technologies Inc., Montreal, Quebec, Canada) and aggrecan (Human Aggrecan EASIA™ ELISA kit, BioSource Europe SA, Nivelles, Belgium) for phenotype expression, the multifunctional peptide transforming growth factor- β1 (TGF-β1, quantikine human TGF-β1 immunoassay, R&D Systems, MN, USA) for anabolic growth factor synthetic activity, interleukin1β (IL-1β, quantikine human IL-1β immunoassay, R&D Systems, MN, USA) and matrix metalloprotease 3 (MMP3, quantikine human MMP3 immunoassay, R&D Systems, MN, USA) for inflammatory and catabolic response assessment, respectively. The measured protein concentrations were normalized by Total Protein concentration (Total Protein Kit, Micro Lowry method, Petterson’s Modification, SIGMA®, Missouri, USA). The cell proliferation reagent WST-1 (Roche Diagnostic GmbH, Penzberg, Germany) test was performed and quantified spectrophotometrically at 450 nm with the reference wavelength at 625 nm. Results were expressed as % Vitality calculated as follows:

$$\%\text{Vitality}=\frac{100\;\times \;\text{OD}\;s}{\text{OD}\;\text{CTR}}$$

where *ODs* was the optical density of the sample and *OD CTR* was the optical density of control wells.

### Histology and scanning electron microscopy

After 14 days of cell culture, the seeded membranes were studied to evaluate chondrocyte and mesenchymal stromal cell colonization inside HDM_derm by histology and scanning electron microscopy (SEM). For histology, specimens were fixed in 10% buffered formalin solution in PBS (pH 7.4), embedded in paraffin and sections were cut to 6-μm thickness and stained with Haematoxilin & Eosin, Safranin O and Alcian Blue. Images were grabbed by using an Olympus BX51 microscope equipped with XC30 Olympus camera. For SEM, specimens were fixed in 2.5% v/v glutaraldehyde (pH 7.4 in PBS 0.01M, 1h) and dehydrated in graded ethanol series. After a passage in hexamethyldisilazane, the samples were air dried, then sputter-coated with gold before being examined by a Philips XL-20 SEM.

### Statistical analysis

Statistical evaluation of data was performed using the SPSS/PC+ Statistics TM 12.1 (SPSS Inc., Chicago, IL USA) software package. Data are reported as Mean ± SD at a significance level of *p* < 0.05. After checking normal distribution and homogeneity of variance, two-way ANOVA (group and experimental time) followed by Bonferroni’s *t* test were used to compare results. Student’s *t* test was used to compare cell vitality index results.

## Results

### *In vitro* model

At 24 hours after seeding, cells adhered consistently to HDM_derm membranes (93% and 98% for NHAC-kn and hMSC, respectively). At 7 days, the cell vitality index was 98% for both cell cultures seeded on HDM_derm. After 14 days of culture, these indexes significantly (*p* < 0.0005) increased for both cell cultures (NHAC-kn 136%, hMSC 263%).

Tables 1 and 2 show the results of NHAC-kn and hMSC cells seeded on the HDM_derm membrane at 7 and 14 days. The phenotypic expression given by the synthesis of CPII did not differ within the two cell types. The synthesis of CPII on HDM_derm increased between experimental times for both cell types (NHAC-kn, *p* < 0.05; hMSC, *p* < 0.05). At both experimental times, HDM_derm induced NHAC-kn to produce almost double the amount of CPII than did CTR (*p* < 0.05). The synthetic activity, expressed by the measured concentration of aggrecan, increased over time in both cells (*p* < 0.0005). NHAC-kn (14 days) and hMSC (7 and 14 days) seeded onto HDM_derm produced significantly higher amounts of aggrecan than those seeded on CTR (*p* < 0.0005). Regarding the synthesis and release of TGF-β1, significant decreases were found for NAHC-kn (*p* < 0.05) and hMSC (*p* < 0.0005) in the CTR group between experimental times. For hMSC, significantly higher TGF-β1 values were found when cultured on HDM_derm than those found on CTR at both 7 and 14 days (*p* < 0.0005). Concerning inflammatory responses, no statistically difference in the release of IL-1β was observed for both cells and experimental times. At 7 days NHAC-kn seeded onto HDM_derm produced significantly lower values of MMP-3 compared with CTR (*p* < 0.005). Furthermore, the MMP-3 released by NHAC-kn in CTR was observed to decrease significantly over the culture time (*p* < 0.005).

**Table 1:** ***In vitro *results of NHAC-kn cultured on HDM_derm and CTR for 7 and 14 days**

	**HDM_derm**		**CTR**		**Two-way ANOVA**
	**7 days**	**14 days**	**7 days**	**14 days**	
CPII (pg/mg)	79.0 ± 13.5*	145.0 ± 21.2*^,^°	39.8 ± 13.8	71.0 ± 7.4	F=6.9, *p* < 0.05
Aggrecan (ng/mg)	490.8 ± 60.0	937.4 ± 76.2***^,^°°°	517.6 ± 32.8	583.4 ± 20.8	F=66.5, *p* < 0.0005
TGF-β1 (pg/mg)	2821.7 ± 145.7	2785.6 ± 405.1	3015.7 ± 125.6	2332.3 ± 89.4°	F=10.2, *p* < 0.05
IL-1β (pg/mg)	0.8 ± 0.1	1.0 ± 0.2	0.8 ± 0.0	0.8 ± 0.0	NS
MMP3 (ng/mg)	5.8 ± 3.8**	3.3 ± 1.5	18.6 ± 1.7	5.8 ± 0.2°°	F=16.7, *p* < 0.005

Bonferroni *t* test (Mean ± SD, n = 8 replicates):

- between HDM_derm and CTR within each experimental time: *, *p*<0.05; **, *p*<0.005; ***, *p*<0.0005.

- between experimental times within each tested membrane: °, *p*<0.05; °°, *p*<0.005; °°°, *p*<0.0005.

**Table 2:** ***In vitro *results of hMSC cultured on HDM_derm and CTR for 7 and 14 days**

	**HDM_derm**		**CTR**		**Two-way ANOVA**
	**7 days**	**14 days**	**7 days**	**14 days**	
CPII (pg/mg)	82.2 ± 1.5	136.8 ± 3.0°	90.9 ± 4.1	140.9 ± 2.6°	F=4.86, *p* < 0.05
Aggrecan (ng/mg)	191.7 ± 6.1***	265.7 ± 7.4***^,^ °°°	129.7 ± 3.5	184.0 ± 5.0°°°	F=24.02, *p* < 0.0005
TGF-β1 (pg/mg)	926.9 ± 4.5***	894.1 ± 18.9***	644.2 ± 23.1	324.1 ± 75.6°°°	F=37.3, *p* < 0.0005
IL-1β (pg/mg)	0.5 ± 0.0	0.5 ± 0.1	0.4 ± 0.0	0.6 ± 0.0	NS
MMP3 (ng/mg)	0.01 ± 0.01	0.18 ± 0.03	Not detected	Not detected	NS

Bonferroni *t* test (Mean ± SD, n = 8 replicates):

- between HDM_derm and CTR within each experimental time: ***, *p*<0.0005.

- between experimental times within each tested membrane: °, *p*<0.05; °°°, *p*<0.0005.

Significant phenotypic marker expression data obtained from NHAC-kn and hMSC cultures seeded on HDM_derm were normalized to those of their controls (Table 3). When comparing normalized data, NHAC-kn showed a significant increase in CPII synthesis with respect to that showed by hMSC cells, approximately 2.2 fold, at both experimental times (*p* < 0.005), whereas hMSC cells induced a significant increase in aggrecan at 7 days (1.6 fold, *p* < 0.005) and TGF-β1 at both experimental times (approximately 1.5 fold at 7 days and 2.3 fold at 14 days) compared with that induced by NHAC-kn cells (*p* < 0.005).

**Table 3:** Normalized data of NHAC-kn and hMSC on HDM_derm to control at 7 and 14 days

	**Experimental time**	**NHAC-kn**	**hMSC**
CPII/CPII _CTR_	7 days	2.02 ± 0.31**	0.90 ± 0.02
	14 days	2.07 ± 0.28**	0.97 ± 0.02 °
Aggrecan/Aggrecan _CTR_	7 days	0.92 ± 0.12	1.48 ± 0.04**
	14 days	1.58 ± 0.13°°	1.45 ± 0.03
TGF-β1/TGF-β1 _CTR_	7 days	0.95 ± 0.05	1.44 ± 0.01**
	14 days	1.22 ± 0.17 °	2.76 ± 0.06**^,^°°

Bonferroni *t* test (Mean ± SD, n = 8 replicates):

- between NHAC-kn and hMSC within each experimental time: **, *p*<0.005.

- between experimental times within each primary cell: °, *p* < 0.05; °°, *p*<0.005.

### Histology and Scanning Electron Microscopy (SEM)

**Figure 1:**
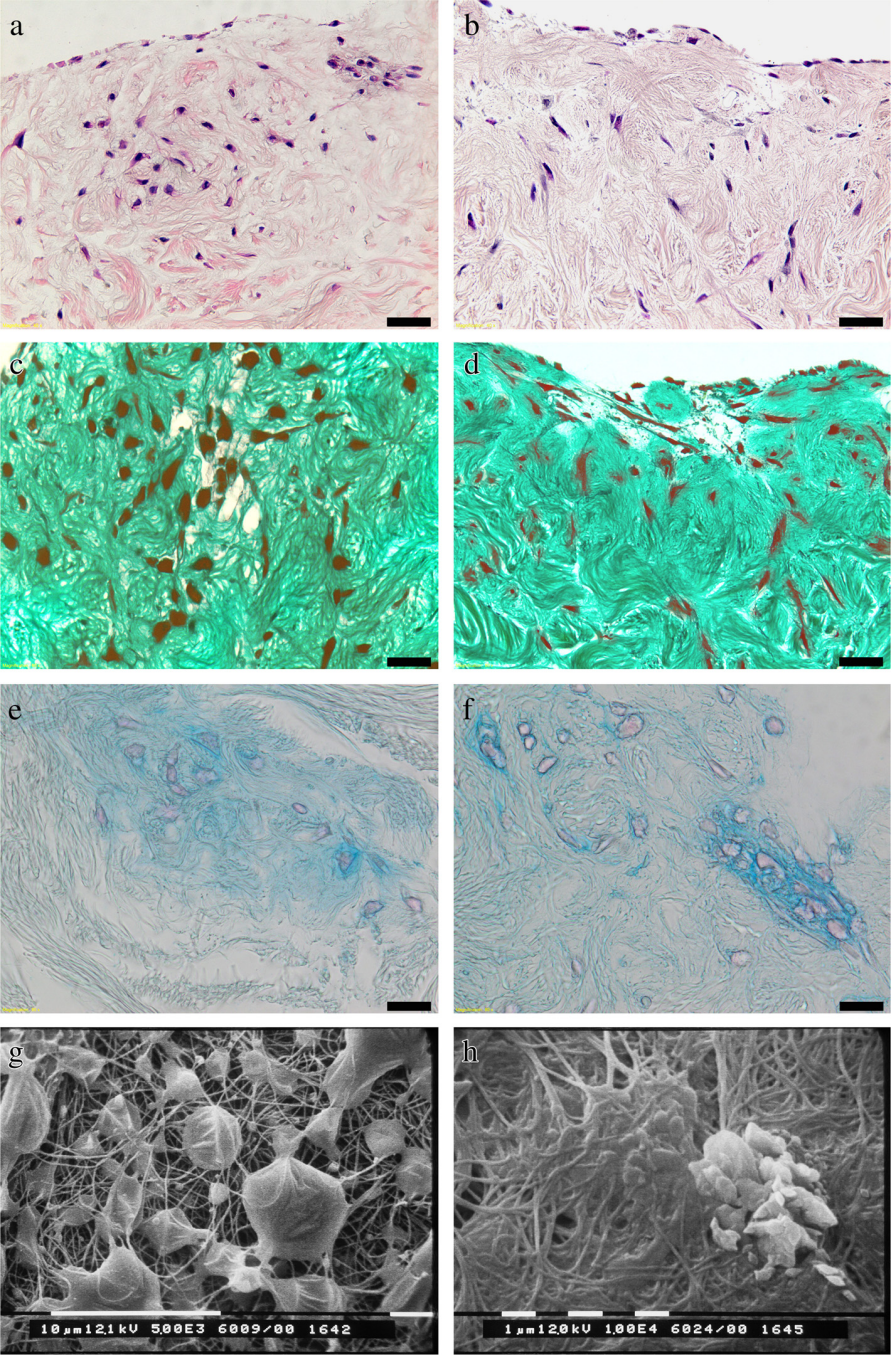
**Histology and SEM patterns of NHAC-kn and hMSC seeded on HDM_derm at 14 days.** Histological and SEM images of NHAC-kn (**a**,**c**,**e**,**g**) and hMSC (**b**,**d**,**f**,**h**). The NHAC-kn and hMSC cells are qualitatively recognizable in HDM_derm. Haematoxilin & Eosin staining (**a**,**b**), magnification of 40x (scale bar = 20 μm): both cell types grew superficially and deeply colonized the HDM_derm. Safranin O staining (**c**,**d**), magnification of 80x (scale bar = 10 μm): presence of proteoglycan synthesis (dark red), predominantly inside cells and equally present in both primary cells. Alcian blue staining (**e**,**f**), magnification of 80x (scale bar = 10 μm): around both primary cells light blue stain indicated the presence of GAGs deposited on the decellularized matrix. SEM images show a complete colonization of HDM_derm scaffold by NHAC-kn with collagen network still visible; NHAC-kn retained their viable morphology and cell-to-cell relationship (**g**). Sparse distribution of the few hMSC cell aggregates is present (**h**).

The main histo-architecture of the acellular extracellular matrix was retained. After 14 days in static culture, both cells grew prevalently on the HDM_derm surface. NHAC showed rounded features forming aggregates along the HDM_derm surface and penetrating in the deeper layers of the decellularized membrane (Figure 1a). hMSC exhibited a fibroblast-like morphology with elongated nuclei covering the membranes, whereas occasional cells were observed to infiltrate more deeply (Figure 1b). Safranin O (Figures 1c,d) and Alcian blue (Figures 1e,f) stainings showed proteoglycan synthesis, only at intracellular level, and the presence of GAGs deposited on the decellularized matrix just in the proximity of grown cells, respectively, without differences between NHAC-kn and hMSC.

SEM analysis performed 14 days after seeding on HDM showed different patterns for NHAC-kn and hMSC, respectively. A homogeneous and multilayered surface-covering colonization with possibly living cells was found for NHAC-kn on HDM_derm: they had a polygonal-rounded morphology or fusiform shape. Most of the cells were connected to each other by forming cell aggregations and were tightly interwoven within the collagen network. Conversely, hMSCs partially covered HDM_derm by cell aggregates associated with a clear-cut collagen meshwork (Figure 1g,h).

## Discussion

The aim of the present study was to assess the influence of HDM_derm on two human primary cells – NHAC-kn and hMSC −, in terms of CPII, aggrecan, TGF-β1, IL-1β and MMP-3 release, in order to evaluate its possible use as a scaffold for *in situ* tissue engineering techniques in cartilage regenerative treatments. Human primary cells were chosen because they provide conditions that closely simulate a living model, thus yielding more physiologically significant results than animal-derived cells or cell lines. Since it is recognized that intra-species variability occurs, reproducibility of results was checked by taking into consideration dermis donor variability and not donor cell variability and thus four different dermis donors were tested.

The current results showed that HDM_derm supported NHAC-kn and hMSC cell adhesion, vitality, chondrogenic differentiation and synthetic activity. The NHAC-kn cultures maintained their phenotype with significantly increased synthesis of CPII and aggrecan, whereas the hMSC cultures showed a significant increase in aggrecan and TGF-β1 secretion. Furthermore, HDM-derm did not induce the release of IL-1β and MMP-3 that are often used as markers to measure inflammatory and catabolic stimuli *in vitro*. The comparison between NHAC-kn and hMSC normalized data revealed that, compared with hMSC, NHAC-kn were able to increase CPII synthesis at both experimental times, whereas hMSC were able to increase aggrecan at 7 days and TGF-β1 at both experimental times in comparison with NHAC-kn. In addition, the increased production of TGF-β1 by hMSC cells over the control wells may be responsible for the increased aggrecan synthesis via autocrine and paracrine pathways, as also suggested by other authors [15,16]. In the present authors’ opinion, the differences observed between the cell-seeded construct and empty polystyrene wells were due to several factors: (1) stage of chondrogenic differentiation, NHAC-kn being already phenotypically differentiated chondrocyte cells; (2) the presence of a bioactive ECM membrane, as an environmental condition that is recognized as being a key factor in regulating cell behavior [17]; (3) the 3D-culture system condition, that is known to affect chondrogenesis in particular.

The reason why HDM_derm retained bioactivity upon decellularization might be because it participates and directly enhances cellular adhesion and proliferation indexes as found with NHAC-kn and hMSC, at least in the early stages of culture. Some authors found differences among growth factors (GFs) such as VEGF, FGF and TGF-β release in the extraction vehicle from different decellularized membranes [5,18,19]. Some TGF-β1 activity was still detected in the extract of the tested decellularized HDM_derm (428.36 ± 58.89 pg/mg) almost at the same concentration as the non-decellularized HDM_derm (417.01 ± 146.16 pg/mg), suggesting that HDM_derm is a bioactive substrate [6]. In the present authors’ opinion, the native retained TGF-β1 is released from decellularized samples in a short culture time (within 6 days), thus affecting cell adhesion and early proliferation [5,6,8,20]. After that, it might be assumed that the anabolic action of native TGF-β1 is almost completely exhausted by replacing medium twice a week and degradation processes, due to its finite lifespan.

Histological and SEM investigations supported *in vitro* data and showed that the HDM_derm was colonized by both cell types. Safranin O staining showed proteoglycans mainly at intracellular level and partially just around cells in both cultures, whereas Alcian Blue staining indicated glycosaminoglycan deposition at extracellular levels. These findings may suggest that, although GAGs have already been secreted by both primary cells, the macromolecules of proteoglycans, consisting of a core protein on which numerous GAG chains are attached, have yet to be assembled.

The experimental set up of the study had some weaknesses. First of all, the lack of a more clinically relevant matrix as a control was a major limitation. The ideal control material for the present study would be a dermis from the same donors decellularized by an already developed and recognized procedure. Unfortunately, these decellularization techniques that are used in the production of clinically accepted products are patented and it was not possible to reproduce this technique on the dermis derived from the donors included in the present study. Therefore, it was preferred to perform experiments without using other materials with different chemical and physical properties such as synthetic scaffolds or hydrogels.

The use of single-cell seeding density and relatively short culture time was the second weaknesses of the current experimental set up. Therefore, the seeding of HDM_derm probably needed an improvement in the *in vitro* culture conditions. In further *in vitro* experiments, the amount of cells seeded on the scaffold should be addressed, to try and increase the number up to 10^6^ or 10^7^ cells/ml, in order to study the influence of cell concentration on improving the building of a cartilage construct. However, the choice of cell density and experimental times was made to prevent irreversible contact-inhibition and senescence processes that affect NHAC-kn cell controls especially and in light of the authors’ previous *in vitro* experience with primary cultures of rat tenocytes seeded onto HDM_derm [14].

A further improvement in the current study set up might be to adopt a dynamic standardized culture condition by using bioreactors able to ensure efficient cell seeding, colonizing the scaffold deeply and also able to reproduce a mechanical stimulus to improve the cartilage-construct differentiation and maturation as well as regulate microenvironmental key factors in promoting chondrogenesis, such as oxygen concentration and pressure, or the presence of proteases [17,21].

Finally, the strengths of the current study were: (1) the use of a new decellularization method on allogenic human dermis that provided a reliable 3D structural and biological scaffold for chondrocytes and MSCs; (2) the use of primary non-transformed human cells that closely simulate a living model, thus yielding more physiologically significant results than animal derived cells.

Various *in vitro* studies have been performed with different kinds of scaffolds with chondrocytes or MSCs for cartilage regeneration and most of them employed synthetic, biological or composite biomaterials such as poly-(lactic-co-glycolic acid) (PLGA), poly (lactic acid) PLA, poly (glycolic acid) PGA, hydrogel, chondroitin sulphate, hyaluronic acid, collagen, agarose, alginate, chitosan, gelatine, fibroin, fibrin glue or hybrid PLGA-gelatin/chondroitin sulphate/hyaluronic acid as cell supports [22-36].

Over the last few years, decellularized xenogenic and allogenic ECMs have started to be investigated for cartilage tissue engineering, because they retain structural and functional proteins and antibacterial activity, which has been shown within degradation products of biological scaffolds composed of extracellular matrix [37]. However, the few studies testing ECMs, such as bovine cartilage [10], porcine adipose tissue [8], or cartilage [11,13], human adipose tissue [12], or cartilage [9,13], investigating mainly the behaviour of chondrocytes or MSCs in terms of proliferation and viability, concluded that ECMs provided suitable 3D substrates not only for the growth of cells, but also for promoting the formation of new cartilaginous engineered tissue. To our knowledge, the current study is the first to address the use of human decellularized ECM as an alternative 3D scaffold for cartilage regeneration. By comparing the present results to previously cited literature [8-13], it is evident that inflammatory stimuli, and anabolic and catabolic synthetic activity are not deeply investigated biological aspects to evaluate a possible influence of ECM on chondrocyte or MSC behaviour. The decellularization technique employed for the present scaffold did not adversely affect the structural integrity and biological activity of the remaining ECM of HDM_derm, thus its mechanical competence was maintained [6,14]. The advantage of the technique, consisting of a combination of trypsin washes and the extremely low dosage of gamma-ray irradiation (about 0.1 kGy), is to avoid the use of strong chemical agents, which might lead to toxic leachables in the decellularized products, and the terminal sterilization process, which are known to be the main causes of distinct host tissue histological and morphologic responses [6,14].

Further *in vitro* studies are mandatory to test the use of HDM_derm with tissue engineering techniques. The present study on the possible application of HDM_derm for cartilage suggests that this derived matrix might be investigated also as a functional scaffold for chondrocyte repopulation for *in situ* engineering techniques. HDM_derm seems to be a suitable matrix for cartilage regeneration, because it has been shown by the present study to maintain cellular viability and differentiation, and to retain structure and bioactivity, particularly TGF-β1 that might counteract catabolic IL-1β effects, whose levels have been found to correlate with the severity of cartilage damage *in vivo*. Finally, all *in vitro* data need to be transferred *in vivo* into specific preclinical validated models of acute, chronic or degenerative cartilage lesions to assess the therapeutic and functional effectiveness of HDM_derm in cartilage repair or regeneration.

## Conclusions

In summary, to the present authors’ knowledge, this study is the first piece of evidence to support the potential use of human decellularized dermal matrix as a scaffold for cartilage regeneration. The results showed that the developed HDM_derm might be used as a scaffold by acting as a biological and bioactive substrate for cell seeding, proliferation and synthetic activity. HDM_derm sustained the growth and colonization of a well-differentiated phenotype NHAC-kn as well as undifferentiated hMSCs. These results support the use of HDM_derm for further preclinical *in vitro* and *in vivo* models for cartilage tissue engineering.

## Abbreviations

CDM: Chondrocyte Differentiation Medium; CGM: Chondrocyte Growth Medium; CPII: Procollagen Type II C-Propetide; CTR: Controls; ECM: Extracellular Matrix; FGF: Fibroblast Growth Factor; GF: Growth Factor; HDM_derm: Decellularized Human Dermis; hMSC: Human Mesenchymal Bone Marrow-Derived Stromal Cells; IL-1β: Interleukin 1 Beta; MMP-3: Matrix Metalloprotease 3; MSCGM: Mesenchymal Stem Cell Growth Medium; NHAC-kn: Normal Human Knee Cartilage-Derived Articular Chondrocytes; SEM: Scanning Electron Microscopy; TGF-β1: Transforming Growth Factor Beta1; VEGF: Vascular Endothelial Growth Factor.

## Competing interests

The Authors EB, DM, RG, MF patented the decellularization method.

## Authors’ contributions

Contributions of the authors to the manuscript included: *Experimental design*: EB, DM, RG, AC, RR, MF; *Culture assays and Acquisition of data*: EB, GC, PT, FV; *Statistical analysis and Interpretation of data*: GG, PT; *Manuscript drafting*: GG, MT, PT, FV; *Manuscript revising*: GG, MT, RG, SP, MF. All authors have read and approved the final manuscript.

## Pre-publication history

The pre-publication history for this paper can be accessed here:

http://www.biomedcentral.com/1471-2474/14/12/prepub
